# 3-Chloro-*N*′-(3-eth­oxy-2-hy­droxy­benzyl­idene)benzohydrazide monohydrate

**DOI:** 10.1107/S160053681100122X

**Published:** 2011-01-15

**Authors:** Tian-Yi Li, Peng-Tao Zeng

**Affiliations:** a School of Chemical Engineering, Changchun University of Technology, Changchun 130012, People’s Republic of China

## Abstract

In the title compound, C_16_H_15_ClN_2_O_3_·H_2_O, the water mol­ecule is linked to the Schiff base mol­ecule *via* an O—H⋯O hydrogen bond. In the Schiff base mol­ecule, an intramolecular O—H⋯N hydrogen bond occurs and the dihedral angle between the two benzene rings is 20.5 (5)°. In the crystal, the Schiff base and water mol­ecules are linked by inter­molecular N—H⋯O and O—H⋯O hydrogen bonds, forming layers in the *ab* plane.

## Related literature

For Schiff base compounds, see: Bessy *et al.* (2006 ▶); Podyachev *et al.* (2007 ▶); Raj & Kurup (2007 ▶); Pouralimardan *et al.* (2007 ▶); Bacchi *et al.* (2006 ▶); Dinda *et al.* (2002 ▶). For reference bond lengths, see: Allen *et al.* (1987 ▶). The title compound was prepared by the method described in Zhu (2010 ▶).
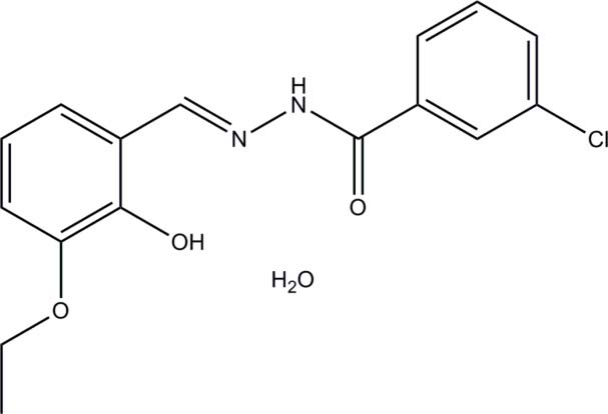


## Experimental

### 

#### Crystal data

C_16_H_15_ClN_2_O_3_·H_2_O
*M* *_r_* = 336.77
Orthorhombic, 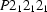

*a* = 4.631 (2) Å
*b* = 13.558 (3) Å
*c* = 25.478 (3) Å
*V* = 1599.7 (8) Å^3^
*Z* = 4
Mo *K*α radiation
μ = 0.26 mm^−1^
*T* = 298 K
0.23 × 0.22 × 0.20 mm

#### Data collection

Bruker APEXII CCD area-detector diffractometer
Absorption correction: multi-scan (*SADABS*; Bruker, 2005 ▶) *T*_min_ = 0.943, *T*_max_ = 0.950
8650 measured reflections
3460 independent reflections
1391 reflections with *I* > 2σ(*I*)
*R*_int_ = 0.085

#### Refinement

*R*[*F* ^2^ > 2σ(*F* ^2^)] = 0.067
*wR*(*F* ^2^) = 0.158
*S* = 1.00
3460 reflections
219 parameters
4 restraints
H atoms treated by a mixture of independent and constrained refinement
Δρ_max_ = 0.20 e Å^−3^
Δρ_min_ = −0.18 e Å^−3^
Absolute structure: Flack (1983 ▶), 1399 Friedel pairs
Flack parameter: 0.25 (16)

### 

Data collection: *APEX2* (Bruker, 2005 ▶); cell refinement: *SAINT* (Bruker, 2005 ▶); data reduction: *SAINT*; program(s) used to solve structure: *SHELXS97* (Sheldrick, 2008 ▶); program(s) used to refine structure: *SHELXL97* (Sheldrick, 2008 ▶); molecular graphics: *SHELXTL* (Sheldrick, 2008 ▶); software used to prepare material for publication: *SHELXL97*.

## Supplementary Material

Crystal structure: contains datablocks global, I. DOI: 10.1107/S160053681100122X/om2396sup1.cif

Structure factors: contains datablocks I. DOI: 10.1107/S160053681100122X/om2396Isup2.hkl

Additional supplementary materials:  crystallographic information; 3D view; checkCIF report

## Figures and Tables

**Table 1:** Hydrogen-bond geometry (Å, °)

*D*—H⋯*A*	*D*—H	H⋯*A*	*D*⋯*A*	*D*—H⋯*A*
O2—H2*A*⋯N1	0.82	1.95	2.645 (5)	142
N2—H2⋯O4^i^	0.90 (1)	2.03 (1)	2.932 (5)	175 (5)
O4—H4*A*⋯O3	0.84 (3)	1.89 (2)	2.717 (5)	167 (5)
O4—H4*B*⋯O2^ii^	0.85 (4)	2.16 (2)	2.945 (5)	154 (4)

Symmetry codes: (i) 
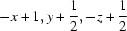
; (ii) 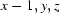.

**Fig. 1.**
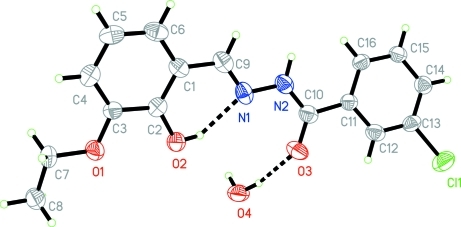
The molecular structure of the title compound with 30% probability displacement ellipsoids for non-hydrogen atoms. Hydrogen bonds are drawn as dashed lines.

**Fig. 2.**
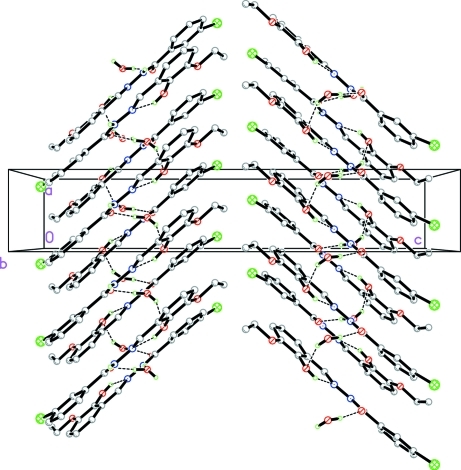
The molecular packing of the title compound. Hydrogen bonds are drawn as dashed lines.

## supplementary crystallographic information

### Comment

In the last few years, a number of Schiff bases derived from the reaction of aldehydes with benzohydrazides were prepared and structurally characterized (Bessy *et al.*, 2006; Podyachev *et al.*, 2007; Raj & Kurup, 2007; Pouralimardan *et al.*, 2007; Bacchi *et al.*, 2006; Dinda *et al.*, 2002). As a continuation of the work, in the present paper, the title new Schiff base compound, Fig. 1, is reported.

The compound contains a Schiff base molecule and a water molecule of crystallization. The water molecule is linked to the Schiff base molecule *via* intermolecular O—H···O hydrogen bonds (Table 1). In the Schiff base molecule, there is an O—H···N hydrogen bond, which contributes to the planarity of the molecule. The dihedral angle between the two benzene rings is 20.5 (5)°. All the bond lengths are within normal values (Allen *et al.*, 1987). The molecules are linked through intermolecular N—H···O and O—H···O hydrogen bonds (Table 1) to form two-dimensional layers along the *ab* plane (Fig. 2).

### Experimental

The compound was prepared and crystallized according to the literature method (Zhu, 2010). 3-Ethoxy-2-hydroxybenzaldehyde (0.166 g, 1 mmol) and 3-chlorobenzohydrazide (0.171 g, 1 mmol) were dissolved in 30 ml 95% ethanol. The mixture was stirred at reflux for 10 min, and cooled to room temperature. The clear colorless solution was left to slow evaporation in air for a week, yielding colorless block-shaped crystals, which were collected by filtration and washed with ethanol.

### Refinement

The amino and water H atoms were located from a difference Fourier map and refined isotropically, with the N—H, O—H, and H···H distances restrained to 0.90 (1), 0.85 (1), and 1.37 (2) Å, respectively. The other H atoms were positioned geometrically and refined using the riding-model approximation, with C—H = 0.93–0.97 Å, and O—H = 0.82 Å, and *U*_iso_(H) = 1.2*U*_eq_(C) or *U*_iso_(H) = 1.5*U*_eq_(C8 and O2).

### Crystal data

**Table:**

C_16_H_15_ClN_2_O_3_·H_2_O	*D*_x_ = 1.398 Mg m^−^^3^
*M**_r_* = 336.77	Mo *K*α radiation, λ = 0.71073 Å
Orthorhombic, *P*2_1_2_1_2_1_	Cell parameters from 705 reflections
*a* = 4.631 (2) Å	θ = 2.6–24.5°
*b* = 13.558 (3) Å	µ = 0.26 mm^−^^1^
*c* = 25.478 (3) Å	*T* = 298 K
*V* = 1599.7 (8) Å^3^	Block, colorless
*Z* = 4	0.23 × 0.22 × 0.20 mm
*F*(000) = 704	

### Data collection

**Table:**

Bruker APEXII CCD area-detector diffractometer	3460 independent reflections
Radiation source: fine-focus sealed tube	1391 reflections with *I* > 2σ(*I*)
graphite	*R*_int_ = 0.085
ω scans	θ_max_ = 27.0°, θ_min_ = 2.2°
Absorption correction: multi-scan (*SADABS*; Bruker, 2005)	*h* = −5→5
*T*_min_ = 0.943, *T*_max_ = 0.950	*k* = −12→17
8650 measured reflections	*l* = −32→29

### Refinement

**Table:**

Refinement on *F*^2^	Secondary atom site location: difference Fourier map
Least-squares matrix: full	Hydrogen site location: inferred from neighbouring sites
*R*[*F*^2^ > 2σ(*F*^2^)] = 0.067	H atoms treated by a mixture of independent and constrained refinement
*wR*(*F*^2^) = 0.158	*w* = 1/[σ^2^(*F*_o_^2^) + (0.0181*P*)^2^] where *P* = (*F*_o_^2^ + 2*F*_c_^2^)/3
*S* = 1.00	(Δ/σ)_max_ = 0.002
3460 reflections	Δρ_max_ = 0.20 e Å^−^^3^
219 parameters	Δρ_min_ = −0.18 e Å^−^^3^
4 restraints	Absolute structure: Flack (1983), 1399 Friedel pairs
Primary atom site location: structure-invariant direct methods	Flack parameter: 0.25 (16)

### Special details

**Table:**

Geometry. All e.s.d.'s (except the e.s.d. in the dihedral angle between two l.s. planes) are estimated using the full covariance matrix. The cell e.s.d.'s are taken into account individually in the estimation of e.s.d.'s in distances, angles and torsion angles; correlations between e.s.d.'s in cell parameters are only used when they are defined by crystal symmetry. An approximate (isotropic) treatment of cell e.s.d.'s is used for estimating e.s.d.'s involving l.s. planes.
Refinement. Refinement of *F*^2^ against ALL reflections. The weighted *R*-factor *wR* and goodness of fit *S* are based on *F*^2^, conventional *R*-factors *R* are based on *F*, with *F* set to zero for negative *F*^2^. The threshold expression of *F*^2^ > σ(*F*^2^) is used only for calculating *R*-factors(gt) *etc*. and is not relevant to the choice of reflections for refinement. *R*-factors based on *F*^2^ are statistically about twice as large as those based on *F*, and *R*- factors based on ALL data will be even larger.

### Fractional atomic coordinates and isotropic or equivalent isotropic displacement parameters (Å^2^)

**Table:**

	*x*	*y*	*z*	*U*_iso_*/*U*_eq_	
Cl1	−0.1754 (4)	0.65889 (10)	0.04444 (6)	0.1007 (7)	
N1	0.7745 (10)	0.8171 (3)	0.26638 (17)	0.0663 (13)	
N2	0.5846 (11)	0.8403 (3)	0.22645 (18)	0.0638 (13)	
O1	1.3750 (8)	0.6775 (2)	0.41318 (14)	0.0680 (10)	
O2	1.0189 (9)	0.6980 (2)	0.33554 (13)	0.0656 (10)	
H2A	0.8935	0.7128	0.3142	0.098*	
O3	0.4905 (10)	0.6809 (3)	0.20923 (14)	0.0907 (13)	
O4	0.4271 (12)	0.5540 (2)	0.29094 (15)	0.0892 (14)	
C1	1.1199 (12)	0.8733 (4)	0.3283 (2)	0.0550 (14)	
C2	1.1601 (12)	0.7804 (4)	0.3510 (2)	0.0541 (13)	
C3	1.3572 (13)	0.7715 (4)	0.3925 (2)	0.0560 (14)	
C4	1.5152 (12)	0.8508 (4)	0.4093 (2)	0.0636 (14)	
H4	1.6523	0.8430	0.4357	0.076*	
C5	1.4701 (14)	0.9427 (4)	0.3869 (2)	0.0714 (17)	
H5	1.5733	0.9970	0.3989	0.086*	
C6	1.2753 (14)	0.9537 (4)	0.3474 (2)	0.0704 (17)	
H6	1.2452	1.0158	0.3329	0.085*	
C7	1.5824 (12)	0.6631 (4)	0.4544 (2)	0.0704 (16)	
H7A	1.5512	0.7105	0.4823	0.084*	
H7B	1.7767	0.6717	0.4409	0.084*	
C8	1.5442 (15)	0.5608 (3)	0.4746 (2)	0.0836 (19)	
H8A	1.3503	0.5527	0.4873	0.125*	
H8B	1.6777	0.5493	0.5028	0.125*	
H8C	1.5801	0.5144	0.4469	0.125*	
C9	0.9160 (13)	0.8890 (4)	0.2864 (2)	0.0655 (17)	
H9	0.8865	0.9525	0.2737	0.079*	
C10	0.4512 (14)	0.7692 (4)	0.1998 (2)	0.0634 (16)	
C11	0.2476 (12)	0.7998 (4)	0.1579 (2)	0.0538 (13)	
C12	0.1420 (14)	0.7273 (4)	0.1251 (2)	0.0660 (16)	
H12	0.1992	0.6622	0.1297	0.079*	
C13	−0.0471 (14)	0.7514 (4)	0.0857 (2)	0.0619 (16)	
C14	−0.1410 (12)	0.8456 (4)	0.0778 (2)	0.0639 (15)	
H14	−0.2718	0.8602	0.0512	0.077*	
C15	−0.0362 (13)	0.9190 (4)	0.1104 (2)	0.0612 (15)	
H15	−0.0954	0.9839	0.1055	0.073*	
C16	0.1542 (13)	0.8968 (3)	0.1499 (2)	0.0568 (14)	
H16	0.2222	0.9468	0.1716	0.068*	
H2	0.569 (12)	0.9055 (11)	0.2203 (18)	0.080*	
H4A	0.437 (12)	0.586 (3)	0.2626 (9)	0.080*	
H4B	0.334 (10)	0.589 (3)	0.3130 (13)	0.080*	

### Atomic displacement parameters (Å^2^)

**Table:**

	*U*^11^	*U*^22^	*U*^33^	*U*^12^	*U*^13^	*U*^23^
Cl1	0.1293 (16)	0.0775 (10)	0.0952 (12)	−0.0177 (11)	−0.0022 (11)	−0.0238 (9)
N1	0.062 (3)	0.088 (3)	0.049 (3)	0.024 (3)	0.009 (3)	0.009 (3)
N2	0.065 (4)	0.066 (3)	0.060 (3)	0.015 (3)	0.004 (3)	0.008 (3)
O1	0.066 (3)	0.062 (2)	0.076 (2)	−0.006 (2)	−0.018 (2)	0.0077 (19)
O2	0.059 (3)	0.063 (2)	0.074 (3)	0.001 (2)	−0.017 (2)	−0.0018 (19)
O3	0.124 (4)	0.061 (2)	0.086 (3)	0.020 (3)	−0.003 (3)	0.018 (2)
O4	0.138 (4)	0.055 (2)	0.075 (3)	0.009 (3)	0.017 (3)	0.004 (2)
C1	0.040 (3)	0.061 (3)	0.064 (4)	0.011 (3)	0.012 (3)	0.006 (3)
C2	0.041 (3)	0.063 (3)	0.058 (3)	−0.002 (3)	0.008 (3)	−0.005 (3)
C3	0.054 (4)	0.051 (3)	0.063 (4)	0.001 (3)	0.001 (3)	−0.004 (3)
C4	0.055 (4)	0.076 (4)	0.060 (3)	−0.006 (4)	0.007 (3)	−0.007 (3)
C5	0.068 (5)	0.057 (4)	0.089 (5)	−0.003 (3)	0.017 (4)	−0.011 (3)
C6	0.071 (5)	0.058 (3)	0.083 (5)	0.010 (4)	0.021 (4)	0.006 (3)
C7	0.063 (4)	0.079 (4)	0.069 (4)	0.001 (3)	−0.022 (4)	−0.003 (3)
C8	0.095 (5)	0.069 (4)	0.087 (4)	−0.007 (4)	−0.028 (4)	0.011 (3)
C9	0.056 (5)	0.076 (4)	0.064 (4)	0.016 (3)	0.012 (3)	0.010 (3)
C10	0.071 (5)	0.060 (4)	0.060 (4)	0.007 (4)	0.015 (3)	0.007 (3)
C11	0.058 (4)	0.051 (3)	0.053 (3)	0.001 (3)	0.008 (3)	0.002 (3)
C12	0.073 (4)	0.056 (3)	0.069 (4)	0.012 (4)	0.013 (4)	0.005 (3)
C13	0.070 (4)	0.052 (3)	0.063 (4)	−0.013 (3)	0.008 (4)	−0.008 (3)
C14	0.064 (4)	0.065 (3)	0.063 (4)	0.006 (4)	0.001 (3)	0.003 (3)
C15	0.066 (4)	0.051 (3)	0.067 (4)	0.000 (3)	−0.008 (4)	0.003 (3)
C16	0.066 (4)	0.049 (3)	0.055 (3)	−0.002 (3)	0.007 (3)	−0.005 (3)

### Geometric parameters (Å, °)

**Table:**

Cl1—C13	1.741 (5)	C5—H5	0.9300
N1—C9	1.281 (6)	C6—H6	0.9300
N1—N2	1.381 (6)	C7—C8	1.491 (6)
N2—C10	1.331 (6)	C7—H7A	0.9700
N2—H2	0.901 (10)	C7—H7B	0.9700
O1—C3	1.381 (5)	C8—H8A	0.9600
O1—C7	1.436 (5)	C8—H8B	0.9600
O2—C2	1.354 (5)	C8—H8C	0.9600
O2—H2A	0.8200	C9—H9	0.9300
O3—C10	1.234 (5)	C10—C11	1.485 (7)
O4—H4A	0.84 (3)	C11—C12	1.379 (6)
O4—H4B	0.85 (4)	C11—C16	1.399 (6)
C1—C6	1.394 (7)	C12—C13	1.371 (7)
C1—C2	1.399 (6)	C12—H12	0.9300
C1—C9	1.440 (7)	C13—C14	1.365 (6)
C2—C3	1.402 (7)	C14—C15	1.384 (6)
C3—C4	1.369 (6)	C14—H14	0.9300
C4—C5	1.386 (7)	C15—C16	1.370 (7)
C4—H4	0.9300	C15—H15	0.9300
C5—C6	1.360 (7)	C16—H16	0.9300
			
C9—N1—N2	116.5 (5)	C7—C8—H8A	109.5
C10—N2—N1	120.4 (4)	C7—C8—H8B	109.5
C10—N2—H2	126 (3)	H8A—C8—H8B	109.5
N1—N2—H2	114 (4)	C7—C8—H8C	109.5
C3—O1—C7	116.4 (4)	H8A—C8—H8C	109.5
C2—O2—H2A	109.5	H8B—C8—H8C	109.5
H4A—O4—H4B	108 (2)	N1—C9—C1	121.2 (5)
C6—C1—C2	119.3 (5)	N1—C9—H9	119.4
C6—C1—C9	118.8 (5)	C1—C9—H9	119.4
C2—C1—C9	121.8 (5)	O3—C10—N2	122.4 (6)
O2—C2—C1	123.9 (5)	O3—C10—C11	120.3 (6)
O2—C2—C3	117.6 (5)	N2—C10—C11	117.3 (5)
C1—C2—C3	118.5 (5)	C12—C11—C16	118.1 (5)
C4—C3—O1	125.0 (5)	C12—C11—C10	117.5 (5)
C4—C3—C2	121.1 (5)	C16—C11—C10	124.4 (5)
O1—C3—C2	113.9 (5)	C13—C12—C11	120.0 (5)
C3—C4—C5	119.8 (5)	C13—C12—H12	120.0
C3—C4—H4	120.1	C11—C12—H12	120.0
C5—C4—H4	120.1	C14—C13—C12	122.3 (5)
C6—C5—C4	120.2 (6)	C14—C13—Cl1	118.4 (5)
C6—C5—H5	119.9	C12—C13—Cl1	119.3 (4)
C4—C5—H5	119.9	C13—C14—C15	118.2 (5)
C5—C6—C1	121.1 (5)	C13—C14—H14	120.9
C5—C6—H6	119.5	C15—C14—H14	120.9
C1—C6—H6	119.5	C16—C15—C14	120.6 (5)
O1—C7—C8	107.5 (4)	C16—C15—H15	119.7
O1—C7—H7A	110.2	C14—C15—H15	119.7
C8—C7—H7A	110.2	C15—C16—C11	120.8 (5)
O1—C7—H7B	110.2	C15—C16—H16	119.6
C8—C7—H7B	110.2	C11—C16—H16	119.6
H7A—C7—H7B	108.5		

### Hydrogen-bond geometry (Å, °)

**Table:**

*D*—H···*A*	*D*—H	H···*A*	*D*···*A*	*D*—H···*A*
O2—H2A···N1	0.82	1.95	2.645 (6)	142
N2—H2···O4^i^	0.90 (1)	2.03 (1)	2.932 (5)	175 (5)
O4—H4A···O3	0.84 (3)	1.89 (2)	2.717 (5)	167 (5)
O4—H4B···O2^ii^	0.85 (4)	2.16 (2)	2.945 (5)	154 (4)

Symmetry codes: (i) −*x*+1, *y*+1/2, −*z*+1/2; (ii) *x*−1, *y*, *z*.
